# Imaging the Dynamic Interaction Between Sprouting Microvessels and the Extracellular Matrix

**DOI:** 10.3389/fphys.2019.01011

**Published:** 2019-08-22

**Authors:** Adam Rauff, Steven A. LaBelle, Hannah A. Strobel, James B. Hoying, Jeffrey A. Weiss

**Affiliations:** ^1^Department of Biomedical Engineering, University of Utah, Salt Lake City, UT, United States; ^2^Scientific Computing and Imaging Institute, University of Utah, Salt Lake City, UT, United States; ^3^Innovations Laboratory, Advanced Solutions Life Sciences, Manchester, NH, United States

**Keywords:** angiogenesis, neovessels, vascular networks, time-series imaging, extracellular matrix

## Abstract

Thorough understanding of growth and evolution of tissue vasculature is fundamental to many fields of medicine including cancer therapy, wound healing, and tissue engineering. Angiogenesis, the growth of new vessels from existing ones, is dynamically influenced by a variety of environmental factors, including mechanical and biophysical factors, chemotactic factors, proteolysis, and interaction with stromal cells. Yet, dynamic interactions between neovessels and their environment are difficult to study with traditional fixed time imaging techniques. Advancements in imaging technologies permit time-series and volumetric imaging, affording the ability to visualize microvessel growth over 3D space and time. Time-lapse imaging has led to more informative investigations of angiogenesis. The environmental factors implicated in angiogenesis span a wide range of signals. Neovessels advance through stromal matrices by forming attachments and pulling and pushing on their microenvironment, reorganizing matrix fibers, and inducing large deformations of the surrounding stroma. Concurrently, neovessels secrete proteolytic enzymes to degrade their basement membrane, create space for new vessels to grow, and release matrix-bound cytokines. Growing neovessels also respond to a host of soluble and matrix-bound growth factors, and display preferential growth along a cytokine gradient. Lastly, stromal cells such as macrophages and mesenchymal stem cells (MSCs) interact directly with neovessels and their surrounding matrix to facilitate sprouting, vessel fusion, and tissue remodeling. This review highlights how time-lapse imaging techniques advanced our understanding of the interaction of blood vessels with their environment during sprouting angiogenesis. The technology provides means to characterize the evolution of microvessel behavior, providing new insights and holding great promise for further research on the process of angiogenesis.

## Introduction

An understanding of the growth and evolution of tissue vasculatures is fundamental to many fields of medicine including cancer therapy, wound healing, and tissue engineering (Carmeliet, 2005). Blood vessels are dynamic structures that provide tissues with oxygen and nutrients, endocrine signals, and immune access (Carmeliet and Jain, 2011). Changes in vascular growth within tissues occur in response to changing metabolic needs and tissue repair. Angiogenesis, the growth of new vessels from existing ones, is dynamically influenced by a variety of environmental factors, including the tissue matrix. For example, growing vessels form adhesions that can transmit force to the extracellular matrix (ECM) and sense forces from the ECM. They also degrade the ECM around them by secretion of proteases and respond to growth factors for directional growth. Lastly, cells communicate *via* paracrine and juxtacrine signaling to regulate angiogenesis. These factors affect neovessel growth and guidance through the tissue stroma.

Despite our relatively deep knowledge of the process of angiogenesis and its regulation, there remains a gap in our understanding of the interactions between growing neovessels and the tissue microenvironment leading to deterministic vascular patterning. Angiogenesis is highly sensitive to chemical and mechanical factors present in the microenvironment, and distinct modes of interaction between vessels and the ECM are largely known from qualitative experiments (Ingber, 2002; Shiu et al., 2005; Li et al., 2005b). However, quantitative measurements of a signal or combination of multiple signals and the corresponding change in vessel behavior, such as migration direction or elongation rate, are difficult to obtain. Chemical signals such as cytokines and proteases are difficult to visualize over space and time near a growing microvessel. Spatiotemporal mechanical signals associated with ECM properties such as structure, composition, and boundary conditions are also difficult to obtain. However, recent studies that used new imaging technologies to capture the interplay of these signals and microvessels have helped elucidate the dynamics of mechanisms modulating angiogenesis.

Several image-based experimental methodologies enable the study of microvascular networks in space and time. Advancements in imaging technologies permit time-series and volumetric imaging of *in vitro* experiments. Time-series imaging involves capturing a sequence of images at defined locations and times, allowing observation of the dynamic evolution of vascular networks, instead of a single “snapshot” of a complex behavior. In microscopy, volumetric imaging acquires pictures of three-dimensional (3D) space by capturing a sequence of two-dimensional (2D) images at spaced focal planes. These volumetric imaging techniques enable extraction of more physiologically relevant information, as vessels reside in 3D environments *in vivo*, and behave differently on 2D substrates (Friedl and Wolf, 2003; Yamada and Cukierman, 2007). Thus, the ability to visualize microvessel growth over 3D space and time has led to more informative investigations of angiogenesis. Furthermore, experiments involving 3D time-series often require custom design and fabrication to devise a system that sustains culture conditions and enables imaging of both microvessels and their environment. Still, the technology offers unique measurements such as rates of growth and changes in network topology that are physiologically significant and are necessary for advancement of our understanding of angiogenesis.

This review describes how time-series microscopy has been used to study the interactions between growing microvessels and the ECM. First, we review mechanical and biophysical factors known to modulate angiogenic growth and the structure of new microvascular networks. Then, we consider proteolysis by neovessels as they degrade their surrounding matrix with matrix metalloproteases (MMPs). Next, we discuss chemotaxis, the migration of microvessels in response to a gradient of cytokines. Then, we look at the effect of stromal cells, including macrophages and mesenchymal stem cells (MSCs), on sprouting microvessels. Finally, we identify future directions for time-series-based research.

## Mechanical and Biophysical Signals

**Figure 1:**
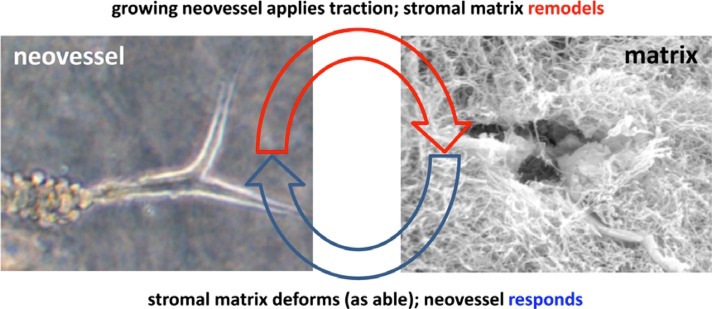
Schematic highlighting the interplay between the growing neovessel and the surrounding matrix structure. Obtained with permission from Hoying et al. (1996).

The development of the microvasculature during angiogenesis is regulated by mechanical and biophysical signals between growing neovessels and their local environment (Ingber, 2002; Rivron et al., 2012). Neovessel sprouts migrate through a complex environment of cells and ECM in the tissue, commonly referred to as the stroma. To advance through stromal matrices, neovessels use both pulling and pushing forces, which reorganize matrix fibers, while contracting and migrating cells can induce large deformations of the surrounding stroma (Shiu et al., 2005; Kirkpatrick et al., 2007; Krishnan et al., 2007; Underwood et al., 2014). Yet, dynamic interactions between neovessels and the stroma are difficult to study with traditional fixed time imaging techniques. Traditional techniques cannot directly observe behaviors such as migration, anastomosis, network elongation, and regression (Mammoto et al., 2009). This is primarily due to the limited number of model systems that permit observation of interactions in 3D space and time under physiologically relevant conditions, and the inherent nonlinearity of the integration of biochemical, biophysical, and mechanical signals (Ingber et al., 1995; Ingber, 2002). Studying the reciprocal relationship (Figure 1) of sprouting neovessels on their surrounding stromal matrix is crucial to determining the morphogenic control of developing vasculature, an important aspect of organ development (Ingber, 2002; Carmeliet, 2005), tissue engineering (Krishnan et al., 2013; Park et al., 2014), and wound healing (Kilarski et al., 2009; Hoying et al., 2014). Biophysical and mechanical factors such as ECM density and stiffness (Shiu et al., 2005; Krishnan et al., 2007; Edgar et al., 2014), fiber orientation (Kirkpatrick et al., 2007; McCoy et al., 2018), and tissue deformation (Underwood et al., 2014) are known to affect sprouting angiogenesis. Time-series microscopy has helped elucidate the dynamic behavior of microvessels in response to these biophysical signals, including the quantification of rates of neovessel elongation and matrix deformation, and new characterization of migratory behavior.

### Effects of Extracellular Matrix Density

**Figure 2:**
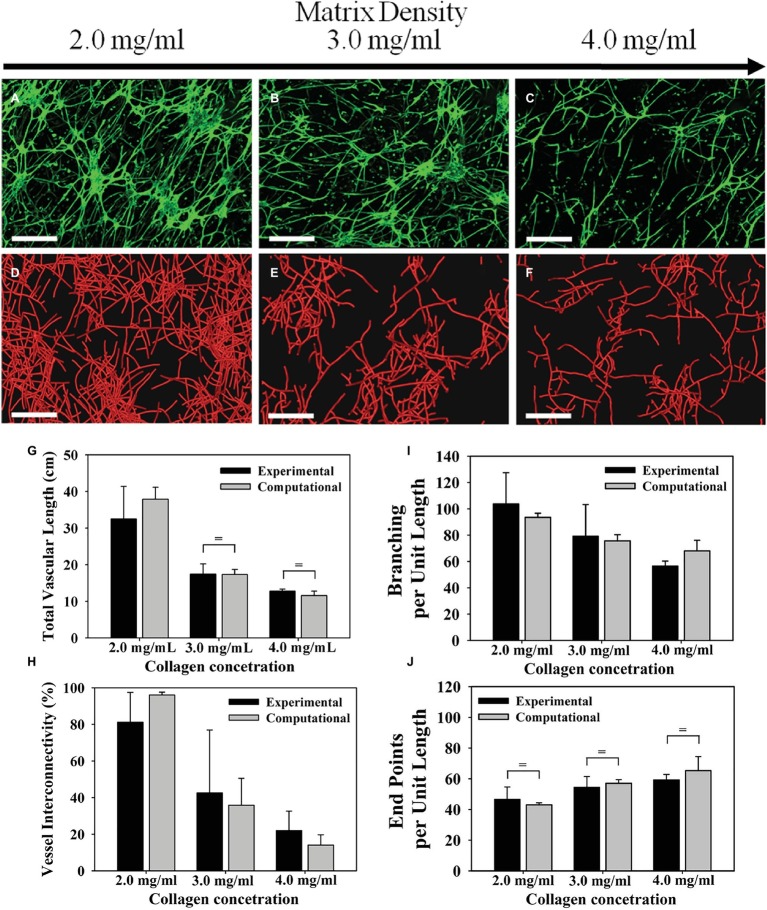
Microvascular networks observed at different levels of collagen density, and associated measurements about the network. Increasing the density of the ECM reduced neovascularization in both the experiments and computational simulations. Top row **(A–C)**: Z-projection mosaic of 3D confocal image data showing vascularized collagen gels taken at Day 6 with different initial collagen concentration. **(D–F)** Results of comparable computational simulations, presented as renderings of the line segment data. **(G)** The total vascular length decreased as matrix density increased. Experimental measurements presented in black and computational predictions presented in gray. **(H)** Vessel interconnectivity, measuring percentage of microvessels that are connected into the largest continuous vascular network, decreased as a function of matrix density. **(I)** Branching point, defined as a node that connected to three or more vessel segments, was created by either a new vessel sprout (branching) or two separate vessels fusing into one (anastomosis). The number of branch points was normalized by the total vascular length to isolate the tendency of microvessels to branch as they grow. Branching per unit length decreased as matrix density increased. **(J)** An end point was defined as a node that was associated with only one vessel segment and represents the terminal end of a vessel. Normalizing the number of end points by the total vascular length revealed that the number of free ends per unit length increased with matrix density. There was a significant effect of matrix density on total vascular length, network connectivity, branch points, and free ends per unit length for both experimental and computational results (one-way ANOVA, *p* < 0.05 in all four cases). Modified with permission from Edgar et al. (2014).

Microvessels are influenced by the density of their ECM substrate, which is related to the stiffness and porosity of the ECM (LaValley and Reinhart-King, 2014). Sieminski et al. found that capillary morphogenesis of human umbilical vein endothelial cells (HUVECs) and human blood outgrowth endothelial cells (HBOEC) is affected by changes of apparent ECM stiffness (Sieminski et al., 2004). Three-dimensional (3D) cultures with type I collagen ECM were subjected to either a change in collagen density or constrained culture edges. At a lower collagen concentration (1.5 mg/ml), vascular networks exhibited greater luminal fractional area and no significant difference between constrained and floating cultures. At higher collagen concentration (3.0 mg/ml), constrained cultures showed decreased luminal fractional area compared with floating cultures. Ghajar et al. embedded HUVECs in varying concentrations of fibrin (2.5–10 mg/ml) and observed that higher fibrin concentrations reduced the growth of capillary networks (Ghajar et al., 2008). However, they also found that the increased density increased stiffness and altered diffusive transport of molecules, which may have contributed to the reduced growth. Edgar et al. used an *in vitro* culture of intact microvessels suspended in type I collagen and a computational model of angiogenesis and found that higher matrix density led to decreased microvessel growth (Figure 2; Edgar et al., 2014). When cultured in type I collagen concentrations of 3–4 mg/ml, microvessels exhibited shorter networks with decreased connectivity compared to a concentration of 2.0 mg/ml. These results were obtained using static measurements extracted from images of a confocal microscope. The vascular network was skeletonized, meaning the fluorescent vessels were segmented and an image processing algorithm was implemented to give a thin version of the vessel network. The skeletons of the network allowed measurement of total vascular length, interconnectivity, branch points, and normalized number of endpoints (Figure 2). The study suggests that for certain density ranges of a particular ECM composition, higher ECM density decreases growth rate, anastomosis, and pruning. Nevertheless, alteration of ECM density is often accompanied by changes in stiffness that can independently affect microvessel growth.

Studying the effects of matrix stiffness and density requires hydrogels with tunable stiffness. Cross-linked collagen gels created by reducing sugar content during polymerization (glycation) have increased stiffness with the same density. Mason et al. embedded spheroids of bovine aortic ECs (BAECs) embedded within type I collagen of varying mechanical properties according to initial ribose concentration. Increasing ECM stiffness independent of density resulted in increased formation of branched cord-like outgrowths from embedded spheroids (Mason et al., 2013). In another study, Bordeleau et al. used an *in vitro* model of BAEC and HUVEC spheroids embedded in type I collagen gel of varying mechanical properties. The study also included an *in vivo* model of tumor angiogenesis, where mice were treated with an inhibitor of the matrix crosslinking enzyme lysyl oxidase to alter tissue stiffness. The study demonstrated that increasing stiffness of the *in vitro* model altered the growth, integrity, and structure of EC vessels structures. Additionally, mice treated with the matrix crosslinking inhibitor had decreased tumor stiffness compared with controls, and the decreased stiffness correlated with significant decrease in the number of microvessel branches (Bordeleau et al., 2017). Alternatively, Sun et al. created hydrogels of varying stiffness and similar densities by adding different concentrations of PEG-dextran to Matrigel. HUVECs were then seeded on the hydrogel surface. The stiffest hydrogels (1,331 Pa) were shown to contain the most dense vascular network with the most branching and smallest cord length, compared to gels of lower stiffness (444 and 8,091 Pa) (Sun et al., 2014). These studies show that increasing ECM stiffness independently of density increases microvessel branching and growth. Nevertheless, studies that increased ECM density, and thereby stiffness, showed decreased microvessel branching and growth (Ghajar et al., 2008; Edgar et al., 2014). This inconsistency could arise because of lack of experimental data, as microvessels have not been observed to grow on stiff enough substrates that cause decrease in vessel growth. Alternatively, both stiffness and density affect microvessel growth in different ways that we do not yet understand. This is partly because previous experiments that observed microvascular network growth quantified global parameters of a vascular network and the entire ECM substrates using images of fixed cultures, which offer limited insight of microvessel behavior.

Imaging microvessel cultures of varying ECM density over time has allowed the observation of evolution of a microvascular network, providing measurements of network regression, maturation and rate of growth. Park et al. used an *in vitro* model with a microfluidic device, time-lapse microscopy, and HUVECs to study the effect of ECM density on microvessel growth. HUVECs were polymerized in type I collagen with initial concentrations varied between 2.0 and 3.0 mg/ml. By measuring regression of vessels, that is the decrease in number of vessel segments over time from 90 to 720 min after seeding, microvessels were found to undergo decreased regression at the lower ECM density. This indicates that lower ECM density is more conductive to formation of a vascular network. Additionally, primitive metrics of network maturation were obtained by measuring the number of connected branches, junctions, and lumen formation of the same microvascular network over 7 days (Park et al., 2014). In another study, Shamloo et al. used a microfluidic device and varied type I collagen concentration between 0.7 and 2.7 mg/ml, while growing human dermal microvascular ECs (HMVECs) on beads. Microvessels exhibited longer sprouts in ECM densities of 1.2 and 1.9 mg/ml, while those grown on higher densities of 2.7 mg/ml formed fewer and shorter viable microvessels sprouts. Using time-lapse imaging, the authors observed the effect of ECM density on sprout elongation and branching over time. This led to the novel observation that HUVEC sprouts formed on beads elongate at a faster rate and undergo more branching events in ECM density ranges between 1.2 and 1.9 mg/ml (Shamloo et al., 2016). These studies were consistent with previous studies observing vascular formation while altering ECM density (Sieminski et al., 2004; Edgar et al., 2014). Even though these studies did not elucidate the effect of matrix stiffness on cellular responses, they introduced novel measurements relevant to neovessel behavior that could not be previously characterized. To understand microvessel response to differential ECM densities and stiffness, we must characterize vascular behavior over time. In addition to density and stiffness, ECM structure affects microvessel growth and evolves in response to vascular growth (Figure 1).

### Microstructural Organization of the Extracellular Matrix

Actively growing neovessels move through the stroma and both reorganize matrix fibers and respond to the matrix structure (Korff and Augustin, 1999; Kirkpatrick et al., 2007; Edgar et al., 2014; Gjorevski et al., 2015). Most stromal matrices are heterogeneous interstitial gels composed of fibrillar collagen, chondroitin sulfates, hyaluronic acid, and other biomolecules (Hughes, 2008; Lu et al., 2011; McGettrick et al., 2012). The reorientation and remodeling of collagen fibrils influences the direction of neovessel migration through a contact guidance mechanism. Korff et al. embedded spheroids of endothelial cells in type I collagen gels, and observed sprouting by two nearby spheroids (Korff and Augustin, 1999). Tensional forces in the matrix generated by the endothelial cell sprouts between nearby cell spheroids remodeled ECM fiber orientation. Additionally, cells from each spheroid sprouted toward each other leading to sprout interactions. This interaction was shown to depend on adhesion to the collagen fibril RGD peptides, as addition of soluble RGD prevented directional sprout growth. In collectively migrating cell populations, similar tension forces resulted in alignment of the matrix fibrils, creating micro-tracks within the matrix. The direction of cell movements correlated with the direction of matrix alignment, indicating that migrating cells directly influence the matrix and exert spatially oriented forces to align matrix elements to navigate through the stroma (Gjorevski et al., 2015).

Substrate microstructural characteristics, including fiber orientation, length, and thickness influence cell behavior (Ingber et al., 1995). Mudera et al. embedded fibroblasts in collagen and exposed them to dual cues of contact guidance and mechanical load (Mudera et al., 2000). Differing degrees of orientation of fibronectin strands provided contact guidance that aligned cells. Cells that were not aligned during the applied loads remodeled their surrounding matrix more rapidly using MMPs (MMP-1, 2, and 3). This suggests matrix remodeling occurs to suit the co-existing mechanical stimuli. In another study, Lai et al. fabricated ECMs of aligned collagen fibrils and cultured human dermal microvascular ECs on aligned and unaligned collagen strips on a glass slide. Migration of ECs on aligned collagen substrates occurred primarily along the direction of fibers, while migration on unaligned substrates occurred randomly in all directions (Lai et al., 2012). Moreover, the shape of the cytoskeleton, as visualized by immunofluorescence, was extended along the direction of the fibers. In addition to directed migration from contact guidance, matrix fiber thickness, and length also influence EC network formation. McCoy et al. embedded human cerebral microvascular endothelial cells (hCMECs) in type I collagen, and allowed collagen polymerization at two different temperatures, 4 or 37°C (McCoy et al., 2016). Hydrogels polymerized in colder conditions contained thicker and longer collagen fibrils. The temperature-induced alteration of fiber size did not influence migration direction. However, polymerization temperature did influence network maturity, as more lumen formation was observed in cold hydrogels.

Sprouting vessels in a 3D environment are assumed to be affected by local fiber orientation of the surrounding ECM, yet experimental observations of 3D matrix fibers by growing neovessels are scarce. Local fiber distribution contributes to the local mechanical response of the vessel microenvironment and the transmission of forces from cell-induced tractions (Guillabert-Gourgues et al., 2016; Hall et al., 2016). Forces from neighboring cells, vessels, or constraints imposed on the tissue boundaries distort the cytoskeleton and surrounding ECM environment (Hall et al., 2016), alter intracellular signaling pathways (Ingber et al., 1995; Mammoto et al., 2009; Rivron et al., 2012), and govern neovessel growth direction and final network shape (Krishnan et al., 2013; Edgar et al., 2014; Underwood et al., 2014). Neovessels growing in denser collagen gels produce less deformation and thus decreased fiber reorganization. Importantly, and consistent with a contact guidance-based mechanism, neovessels grow slower in these high-density matrices (Edgar et al., 2014). Furthermore, neovessels actively remodel their environment as tip cells actively contract, compact, and reorganize matrix structures (Rivron et al., 2012; Gjorevski et al., 2015). The extent of ECM deformation depends on vascular traction and substrate material properties, and also on the distribution of matrix fibers, which in turn influence neovessel growth and organization (Krishnan et al., 2007; Rivron et al., 2012; Underwood et al., 2014). Notwithstanding, physiological mechanisms involved with microvessel migration through 3D matrices remain incomplete. Previous experiments using traditional, fixed-culture methods report observations of an entire vessel network at one time point, rather than evolution of individual microvessels. While ECM structure, including fiber orientations, thickness, and length are known to affect microvessel growth, characterizing ECM microstructure during microvessel behavior remains a challenge. To study the dynamic interaction between microvessels and the structure of their microenvironment we need to observe the evolution of ECM structures along with sprouting angiogenesis.

**Figure 3:**
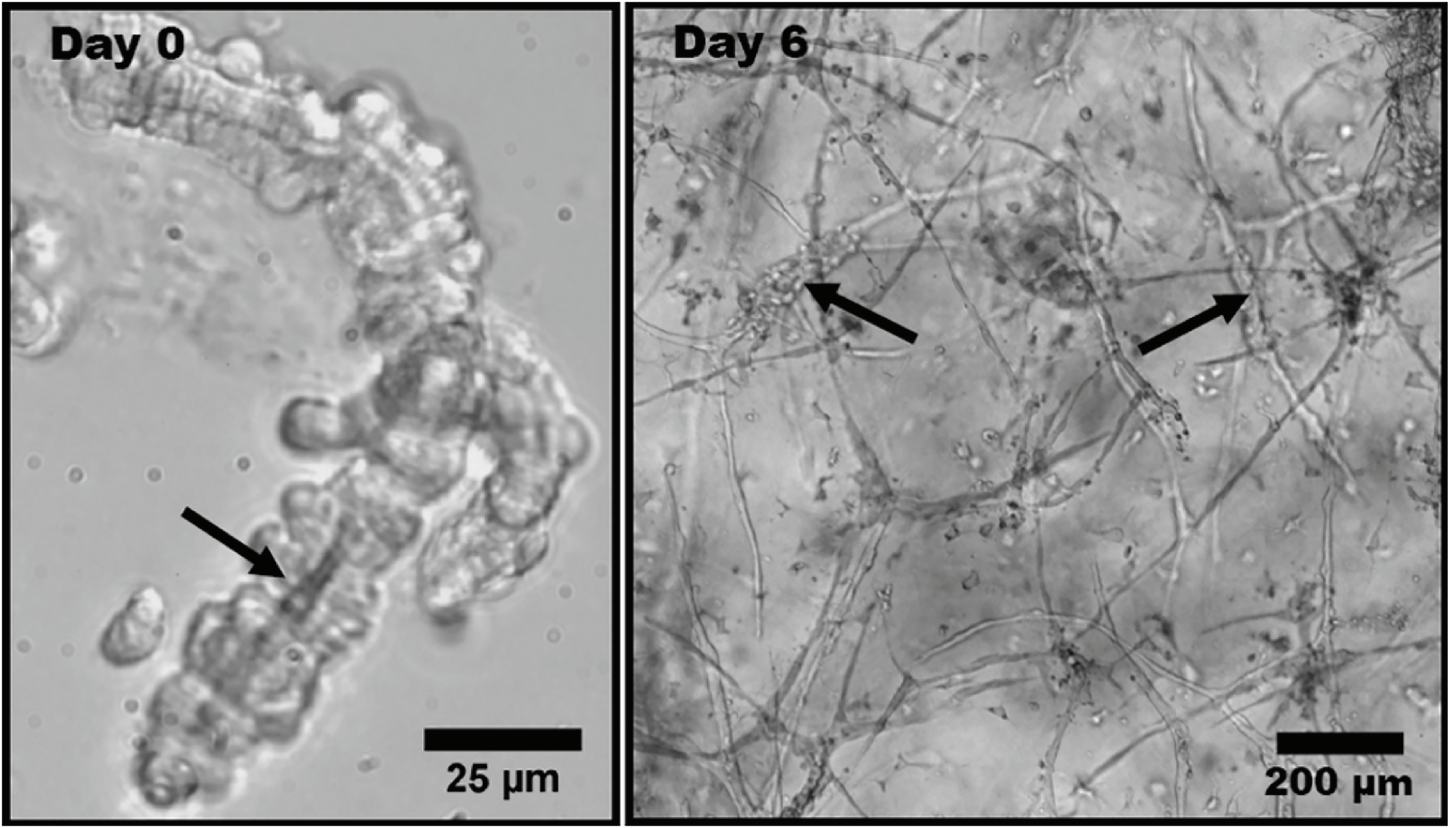
Phase contrast light micrographs of rat microvessel fragments embedded in type I collagen matrix. This experimental model is used to represent angiogenesis *in vitro*. (Left) Isolated microvessel fragment at Day 0 with a visible lumen, indicated by the arrow. (Right) Angiogenic microvessel fragments at Day 6 of growth. The thicker initial fragments are indicated by the arrows. The thinner protrusions extending from the initial fragments are neovessels formed through angiogenesis. Obtained with permission from Edgar et al. (2014).

**Figure 4:**
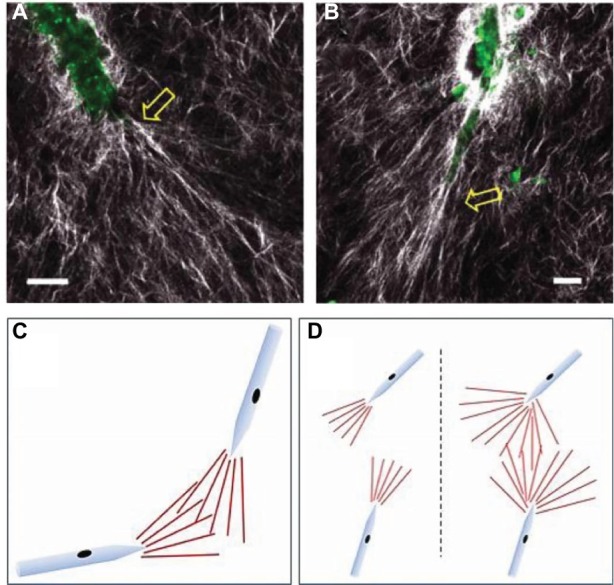
Experimental images and schematics of collagen fibril reorientation by the tips of growing neovessels. **(A,B)** Collagen fibrils (white) and endogenous endothelial cell fluorescence were visualized using SHG/two-photon microscopy. Scale bar = 20 μm. Yellow arrows point to new sprouts arising from the parent microvessel (green). **(C,D)** Schematics of collagen fibril orientation by neovessel tips, and potential mechanism of contact guidance. The “fan” of aligned matrix fibrils (red lines) in front of each growing neovessel would act to “track” the neovessels toward each other when overlapping. **(D)** Consequences to neovessel location due to differences in the size of the fibril-alignment zone. A small fibril-alignment zone would not readily overlap, while a larger zone (due to a less stiff matrix) would make overlapping of these tracks between neighboring neovessels more likely. Modified with permission from Edgar et al. (2014).

Time-lapse imaging allows quantification of active ECM fiber orientation simultaneously with neovessel behavior during angiogenesis. Kirkpatrick et al. used second harmonic generation (SHG) imaging, a nonlinear microscopic imaging technique that can image collagen without use of exogenous labels (Campagnola et al., 2001), to noninvasively visualize changes to fibril organization around angiogenic sprouts and growing neovessels in real time (Kirkpatrick et al., 2007). The investigation utilized an *in vitro* model of angiogenesis that uses intact vessel fragments that recapitulate all aspects of *in vivo* sprouting angiogenesis (Figure 3; Hoying et al., 1996). Higher densities of collagen fibrils formed preferentially around microvascular segments. Increases in collagen fibril density occurred simultaneously with neovessel growth. By using time-lapse imaging, preferential fibril formation around microvessels was demonstrated to be a biologically active event. Moreover, microvessels exerted contractile forces on their surrounding environment that induced local fiber alignment, exhibiting a fan-like structure (Figure 4). The alignment of fibrils by neovessels was quantified by measuring fibril orientation distributions over multiple days in a region adjacent to neovessel tips. Neovessels were found to significantly modify ECM fiber structures throughout the culturing period data. In a later study, McCoy et al. used two different EC types, hCMECs and HUVECs, to study the effects of collagen fiber orientation on vascular formation (McCoy et al., 2018). Varying levels of pre-strain were applied to microwells filled with type I collagen to create alignment of collagen fibrils prior to cell seeding. The results demonstrated that aligned collagen hydrogels promote aligned and thicker microvascular networks with elevated deposition of type IV collagen. Time-lapse imaging was implemented using a bright field microscope placed inside a conventional culturing incubator. Vessel sprouts aligned along pre-existing microstructure. Moreover, when used with pharmacological inhibitors of cell mechanosignaling, inhibition of focal adhesion kinase and myosin II was shown to disrupt the ability of ECs to align along the fibrils. However, inhibition of Rho-associated protein kinase had a significantly decreased effect, a trend that was not observed in cultures with unaligned ECM. This suggests that distinct intracellular signaling pathways of mechanotransduction are involved. These studies characterized biologically relevant information of microvessel growth along with adjacent ECM microstructure. Previously, microstructural characteristics of the fibrous ECM were known to give rise to deterministic microvascular patterning. Using time-lapse imaging we could record the evolution of microvessels and their surrounding stroma. These observations are crucial to the advancement of knowledge on microvessel migration, as it offers direct inspection of 3D migration that could not be investigated previously.

### Forces and Deformation of the Extracellular Matrix

Traction forces applied to the ECM by microvascular endothelial cells modulate vascular growth and capillary morphogenesis (Sieminski et al., 2004; Kniazeva and Putnam, 2009). As neovessels migrate, they form ECM adhesions and use these connections with the ECM to exert traction on the ECM components, resulting in local deformation of the microenvironment (Jakobsson et al., 2010). Migrating neovessels locally degrade the ECM by proteolysis, synthesize ECM and basement membrane, and create matrix deformations by exerting traction forces (Korff and Augustin, 1999; Krishnan et al., 2007; Lee et al., 2009; Edgar et al., 2014; Underwood et al., 2014).

Neovessel tractional forces and matrix stiffness have been studied by imposing fixed boundary conditions on the edges of microvessel cultures. Sieminski et al. cultured ECs on type I collagen in a floating culture by loosening gels from their wells using a spatula (Sieminski et al., 2004). Constrained gels that remained attached to their wells exhibited vessel-like structures with greater accumulation of actin stress fibers. All ECs formed vessel-like structures, but constrained cultures exhibited different network morphologies characterized by shorter total vascular length, smaller vessel-like structures, decreased branching, and larger lumens. Underwood et al. seeded intact microvessels in 3D collagen gels and investigated several different constraint conditions on the boundaries of gels with rectangular and circular cross-sections (Underwood et al., 2014). Traction forces imposed by growing neovessels caused compressive strain that led to realignment of ECM fibers, and large deformation of the entire gel. Hence, cellular traction forces can deform the ECM, realign collagen fibrils, and result in anisotropic material properties for the matrix. Measurements of microvessel network topology, including total vascular length, segment length, and branch points, were quantified by skeletonizing 3D confocal images. Sprouting neovessel growth was demonstrated along a direction perpendicular to compressive strain. Thus, the ability of the sprouting neovessels to deform the matrix resulted in realignment of collagen fibrils, and the neovessels then grew along these aligned fibrils, creating a highly aligned microvascular network.

Neovessel tractions on the ECM can also be studied by confining gel substrates to specific shapes. Nelson et al. seeded bovine pulmonary artery ECs on islands of fibronectin that adhered to a glass slides in circular, rectangular, and square shapes (Nelson et al., 2005). On the circular islands, ECs were quiescent except at the perimeter, where cells continued to proliferate. ECs grown on square islands proliferated toward the island corners with higher preference. Hence, proliferation showed a dependence on substrate geometry, but it also correlated with areas of high stress, as the perimeter of a circle and corners of a square have higher effective local stiffness because of their shapes. Inhibition of molecules that participate in signaling pathways of cellular traction, including Rho kinase (ROCK) and myosin light chain kinase, reduced proliferation. Thus, the study suggests cytoskeletal generated forces are associated with proliferation. In a different study, Sun et al. cultured HUVECs on Matrigel substrates of simple shapes including circle, square, star, and triangle (Sun et al., 2014). The HUVEC networks had longer segments in the center of the substrate compared with areas closer to the substrate boundary for all shapes. Cellular traction forces were also modulated by inhibiting ROCK, which significantly decreased the length of vessel-like segments. This finding suggests that vessels closer to the feature boundaries encounter higher matrix stiffness, and exert greater forces to elongate, migrate, and create a network. Collectively, these studies investigate tractional forces by probing individual proteins associated with the entire culture, measuring deformation of whole gels, or fabricating substrates of confined shapes. These are global measurements that reflect entire vascular networks, but these measurements do not reveal information about local interaction of vessels with the ECM.

**Figure 5:**
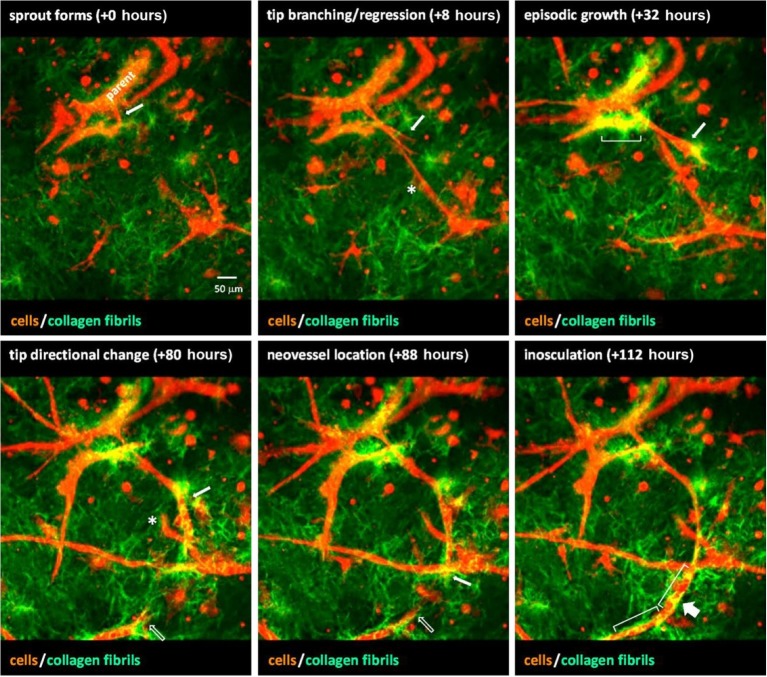
A sequence of images from time-lapse video of neovessel sprouting, growth, and inosculation within a collagen gel stroma. Microvessels (red) were imaged *via* confocal microscopy and collagen fibril structure (green) was visualized using SHG imaging. Over the course of ~4.5 days, a neovessel sprout (white arrow) forms, grows, and changes direction to eventually inosculate (wide arrow) with a second neovessel (open arrow) that appears from out of the field of view. Brackets indicate areas of collagen condensation occurring at neovessel walls. The asterisk marks a neovessel sprout that forms and then regresses approximately 3 days later. Obtained with permission from Edgar et al. (2014).

Time-lapse imaging enables tracking of individual vessel behavior during interaction with the surrounding environment, allowing quantification of matrix deformations over time. The forces exerted by the vessels are difficult to measure, but deformations of a local environment can elucidate information about the induced stress. Kniazeva et al. fabricated an experimental system of cultured ECs on microcarrier beads embedded in fibrin gels. Using this system, the authors observed that higher matrix densities impeded sprouting (Kniazeva et al., 2012). Using time-lapse imaging, the authors further observed the extent of sprouting increased with the rate ECs deformed the fibrin fibers (Kniazeva et al., 2012). This offers direct evidence that neovessel migration and elongation are associated with traction on the ECM. Moreover, the dependence on the rate of deformation means that the cellular induced forces may need to be balanced with the mechanical response of ECM for angiogenic sprouting. In another study, Utzinger et al. used an *in vitro* model of angiogenesis where microvessel fragments were suspended in type I collagen, and SHG imaging was used to investigate microvessel-ECM interactions (Utzinger et al., 2015). Dynamic 3D multiphoton imaging of the gels was performed hourly over 3 days. The study demonstrated that sprouting neovessels dynamically interact with the ECM to deform their surroundings and episodically switch between elongation and regression (Figure 5). Distinct collagen fibril structures were observed around tip cells, suggesting greater traction forces by advancing tips. Du et al. suspended ECs in type I collagen mixed with beads (Du et al., 2016). By tracking beads over time, deformation fields were resolved around the sprouting tips of neovessels. The use of time-lapse imaging, vessel tracking, and deformation field evolution led to a new characterization of microvessel migratory behavior. As a vessel advances it exhibits a “pull,” relating to contractile forces transmitted by adhesion complexes, “release,” “protrusion-related push,” and “retraction-related push.” These studies demonstrate dynamic interaction of growing neovessels with their surroundings that cannot be observed using traditional techniques. Additionally, they offer information about individual microvessels as they navigate the ECM, rather than global parameters of the entire vascular network. The findings from these studies offer new paradigms of sprouting, as forces imparted on the ECM during sprouting involve rates of deformation, local deformation fields, and fiber structures adjacent to microvessels.

## Proteolysis

Proteases play several critical roles in the process of angiogenesis. These roles include degradation of the basement membrane that surrounds the vessel, degradation of the fibrin gel that is leaked from the vascular bed, degradation of the ECM in locations where new vessels are sprouting, creation of space for the new vessels to grow, and release of matrix-bound cytokines or membrane-anchored cytokine receptors. It is important to maintain a balance of protease secretion, as too much matrix degradation can also impair cellular migration (Bacharach et al., 1998) and degrade the mechanical properties of the extracellular matrix (Krishnan et al., 2007).

During angiogenesis, quiescent ECs take on an invasive phenotype and produce proteases that locally degrade their basement membrane at the location of sprouting. Specifically, MMPs MMP-2 and MMP-9 are fundamentally important for this process (Bergers et al., 2000; Fang et al., 2000). MMPs are produced by ECs, and target the major components of the basement membrane, including type IV collagen, type XV collagen, type XVIII collagen, laminins, and heparin sulfate proteoglycans (Roy et al., 2006). MMP production is regulated by growth factors such as VEGF and FGF (Lamoreaux et al., 1998; Zucker et al., 1998; Partridge et al., 2000; Ardi et al., 2009), although MMPs are in turn able to regulate the functionality and availability of these growth factors.

In addition to degrading the basement membrane, ECs must navigate through the ECM, which is primarily composed of Types I and III collagen, elastin, and proteoglycans, depending on the particular tissue type. MMP-2, MMP-9, and MT-MMP-1 (MMP-14) are produced by ECs during angiogenesis (Taraboletti et al., 2002), and they all target the major collagens found in the ECM (Roy et al., 2006). The degradation of ECM can release matrix-bound growth factors that may be necessary for stimulating sprouting and vessel growth (Houck et al., 1992; Plouet et al., 1997). Proteases can also regulate angiogenesis by degrading growth factors and diminishing their activity (Ito et al., 1996).

A handful of innovative methods have been developed for imaging protease activity, and there are several excellent review articles and protocols that describe the methods for imaging of protease activity and proteolysis (Sameni et al., 2000; Jedeszko et al., 2008; Moin et al., 2012; Chalasani et al., 2017). In general, protease activity can be detected by using probes that are based on either monitoring the activity of proteases on the substrate, or by probes that inhibit the protease by binding covalently to the active site of a protease (activity-based probes, or ABPs). For both approaches, a fluorogenic probe is commonly used for detection, although other options have been used such as chromogenic substrates (Van Noorden, 2010). Fluorogenic substrates often use quenched substrates, which combine a nonfluorescent quencher held in close proximity to a reporting fluorophore by a linker peptide that can be degraded by proteases. After degradation of the linker, the reporting fluorophore is separated from the nonfluorescent quencher, yielding a fluorescent signal. A second approach for fluorescent substrates is the use of proteolytic beacons (McIntyre and Matrisian, 2009). These protease substrates use fluorescence resonance energy transfer (FRET) to provide detection due to protease activity (e.g., Scherer et al., 2008). ABPs report the active sites of specific enzymes rather than the proteolytic activity (Blum et al., 2005). Since these probes bind to the active site of the enzyme, they essentially inactivate the enzyme at the time of reporting. All of these approaches to visualizing active enzymes or proteolytic activity are amenable to confocal, multiphoton, and structured illumination microscopy. Live imaging experiments are needed to assess changes in protease activity over time.

Only a handful of studies have examined the distribution of active proteases or protease activity in the context of neovessel growth during angiogenesis, and we found only one study that used true time-series imaging of protease activity during angiogenesis. Most of the proteolysis assays that are available for live imaging have been developed for the study of tumor invasion and metastasis, and thus most of the applications in the literature are related to cancer cells. One of the first studies to use quenched substrates to image proteolysis in living cells was published in 2000 (Sameni et al., 2000). The investigators focused on the study of living human breast cancer cells and used quenched fluorogenic substrates, specifically DQ-BAS or DQ-collagen IV (Thermo Fisher Scientific, Waltham, MA). The quenched substrate was mixed with gelatin and used to coat class coverslips. Cells were plated and observed over 48 h for fluorescence, representing proteolytic degradation of the fluorogenic substrates, using a laser scanning confocal microscope. Finger-like pits or tunnels were observed in the gelatin under the cells at the sites of fluorescence. Although the study reported that time series imaging was used, only a single set of images were presented, acquired 48 h after seeding. A number of subsequent studies were published by the same investigative group, applying the technique to the study of colorectal, prostate, and endothelial progenitor cells (Sameni et al., 2003; Cavallo-Medved et al., 2005; Podgorski et al., 2005; Urbich et al., 2005). All of these studies used single timepoint imaging, typically 48 h after the start of culture.

In recent years, a major focus in angiogenesis research has been the influence of other cell populations on EC sprouting. This has included the study of primarily MSCs and stromal cells. Ghajar et al. examined the mechanisms by which MSCs stimulate capillary morphogenesis from endothelial cells in 3D culture (Ghajar et al., 2010). They used ECs cultured on microcarrier beads, and these were mixed with MSCs in a 3D fibrin matrix. The 3D constructs were doped with a small amount of DQ Collagen I at day 7 prior to imaging. ECs cultured alone resulted in a focal appearance of the fluorescence signal from the DQ collagen substrate. By contrast, EC-MSC cultures showed a very strong fluorescence signal primarily associated with the ECs. RT-PCR revealed that the primary differences in protease message between ECs cultured alone and EC-MSC cultures was increased expression of MT3-MMP and MM-9.

We were only able to find a single study that incorporated time-series imaging of proteolysis during angiogenesis. The study focused on understanding the role of compartmentalization of proteases to caveolae and the dynamics of ECM degradation (Cavallo-Medved et al., 2009). Among other assays, the investigators performed live cell proteolysis assays in 4D during tube formation by endothelial cells. HUVECs were seeded onto DQ-Collagen IV and imaged every 10 min over 16 h. The time series imaging demonstrated that by 4 h the cells had started to align and form small tubular networks. Notably, degradation products of the DQ-Collagen IV were visible pericellularly. By 14 and 16 h, degradation products were observed adjacent to the tubular structures, and at the rear of one of the cells that was migrating toward a developing tubular structure. This study demonstrates the unique ability of fluorogenic substrates to both localize degradation and subsequently track degradation products.

## Chemotactic Factors

**Figure 6:**
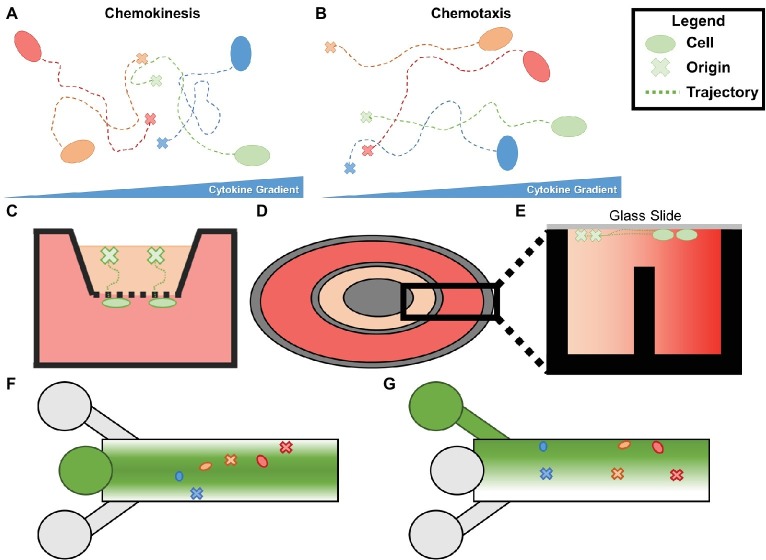
**(A)** Chemokinesis is increase in cellular movement proportional to cytokine concentration without oriented trajectories. **(B)** Chemotaxis is an increase in orientation of a cell’s trajectory in the presence of a cytokine gradient. **(C)** Boyden (transwell) chambers. The bottom chamber containing the chemoattractant and the top chamber containing the cells of interest are separated by a permeable membrane which can be removed and fixed to analyze chemotaxis. **(D)** Top view of a Dunn chamber. **(E)** Profile of Dunn chamber. The outer well containing chemoattractant and the inner well containing controlled media are separated by a central post, allowing a gradient to form between the two. Cells are seeded on glass slides flush with the innermost and outermost rings. **(F)** Simplified schematic of typical microfluidic chemotaxis chamber. A central source well (center left) is flanked by sink channels which form a gradient of chemoattractant from the centerline. Cells are seeded downstream and migrate to the centerline during chemotaxis. **(G)** Alternative schematic in which the top inlet contains the chemoattractant, the middle contains cells and media, and the bottom is a sink.

Angiogenesis is initiated and regulated by a host of soluble and matrix-bound growth factors. The coordination and phenotypic regulation of ECs in growing neovessels are controlled in part by chemotaxis, the growth of cells along a cytokine gradient. Chemotaxis is distinguished from chemokinesis, which is a change in migratory behavior due to the concentration of a cytokine-independent of spatial heterogeneity (Figures 6A,B). *In vivo*, cytokine gradients are formed by cellular secretions, interstitial flow, and differential sequestration of growth factors by ECM proteins. Angiogenic activity progresses over temporal scales that vary from minutes to days and spatial scales that span from the intracellular space to the microvascular network ensemble. Commonly studied angiogenic growth factors include vascular endothelial growth factor (VEGF), fibroblast growth factor (FGF), platelet-derived growth factor (PDGF), tumor necrosis factor alpha (TNF-α), transforming growth factor beta (TGF-β), and angiopoietins (Ang). Angiogenic growth factors participate in numerous signaling pathways including VEGF, Delta-Notch, Rho Rac, and Wnt signaling.

Several types of chemotaxis assays have been used to study the role of chemotaxis in angiogenesis. One of the earliest reported chemotaxis assays is the Boyden (transwell) chamber (Boyden, 1962), and it remains as one of the most commonly used assays for chemotaxis. The assay consists of a reservoir containing chemoattractants separated from a reservoir containing the cells of interest by a permeable membrane (Figure 6C). The permeable membrane is fixed after cell migration to determine the number of cells crossing the membrane. Endpoint chemotaxis assays such as the Boyden chamber do not distinguish chemotaxis from chemokinesis, describe cellular trajectories, or capture the evolution of morphogenic events associated with the development of the microvascular network. Thus, they provide limited information about microvessel growth in response to a cytokine gradient. Time-lapse imaging provides time-series descriptions of cellular trajectories and behavior that can determine the effects of chemotaxis on isolated ECs and microvasculatures. This section reviews early attempts to study the effects of chemotaxis during angiogenesis using time-lapse imaging and more recent methods that allow for qualitative determination of spatiotemporal gradients of soluble and matrix-bound cytokines. Several excellent review articles can be consulted for specific details of chemotaxis assays (Keenan and Folch, 2008; Chung and Choo, 2010; Lee et al., 2018).

### Studies of Chemotaxis Using Chambers and Transwell Migration Assays

Both single cell (e.g., HUVEC studies) and sprouting angiogenic assays (e.g., retina, microvessel studies) have been used to gauge cellular and microvascular responses to chemotaxis *in vitro*. Although these methods do not provide fine control over gradient formation and evolution, they have revealed interactions between growth factors, target cells, cell receptors, and signaling network members.

Commercially available Boyden chamber assays (and transwell migration assays) have been used to determine chemotactic behaviors of ECs. For these assays, a top chamber containing ECs is separated from a chamber containing a chemotactic factor by a permeable barrier, allowing soluble gradients to form between the chambers (Figure 6C; Boyden, 1962). Quantitative counts of cells that pass through the membrane can be made at the end of the assay, but quantitative measurements of cellular trajectories and gradient distribution are not available. Further, gradients within the assay equilibrate in approximately the same time period that it takes for ECs to migrate through the membrane. Thus, the gradient magnitude decreases during the time period that is used to collect migration data until it becomes negligible (Zantl and Horn, 2011). To overcome these limitations, bridge chambers, for example, the Zigmond (rectangular) (Zigmond, 1977) and Dunn (concentric) (Zicha et al., 1991) chambers, were developed (Figure 6D). The improved chambers connect the cell-containing chamber and the cytokine-containing chamber by a thin bridge, with the top of the chamber covered by a glass slide to allow time-lapse imaging (Figure 6E). In one study, HMVECs seeded in the center of a Dunn chamber were imaged every 5 min for 9 h in the presence of bone morphogenic protein 2 (BMP-2), VEGF, pleiotrophin (PTN), a synthetic thrombin mimic (TP508), or control media (Li et al., 2005a). Cells cultured in control media migrated randomly. By contrast, cells cultured in each of the cytokines grew toward the cytokine gradients. Using time-lapse imaging, migration speed was measured, and TP508 was found to induce the highest HMVEC speed of all cytokines. Although the cytokine concentration along the gradients is unknown in bridge chamber assays, they provide a simple method to compare cellular behavior in response to various cytokines.

*In vitro* and *in vivo* assays for angiogenic sprouting can result in gradients in cytokines due to autocrine signaling. Investigators have developed novel ways to eliminate these gradients to study baseline chemotactic behaviors. For instance, soluble fms-like tyrosine kinase-1 (sFlt-1) is a receptor that sequesters VEGF from ECs. In a time-lapse study of an aortic ring sprouting assay, sFlt-1 was used to remove VEGF gradients that developed from the aortic ring in the absence of externally added cytokines (Gerhardt et al., 2003). Filopodia were found to retract after treatment with sFlt-1. By contrast, there was not any filopodia retraction when cultures were treated with VEGF receptor-IgG fusion proteins, which creates dimerized Flt that cannot bind VEGF. Time-lapse imaging allowed measurement of vascular migration and growth, including filopodia and lamellipodia activity, tip cell migration, and sprout elongation, which all decreased in the presence of sFlt-1. Stalk cell proliferation, however, remained unchanged. This information in combination with results from non-time-lapse studies, helped distinguish tip and sprout behavior in the absence of autocrine gradients. In particular, tip cell migration relies on VEGF gradients, while proliferation is a concentration-dependent activity characteristic of stalk cells. Thus, precise gradient control and quantification is not necessary to obtain time-lapse chemotaxis data capable of delineating tip cell migration and stalk cell proliferation.

### Studies of Soluble Chemotaxis Using Microfluidic Devices

The advent of microfluidic devices has permitted quantification and precise, reproducible spatiotemporal control of soluble cytokine distributions. Generally, cells are seeded in a channel that may be coated with matrix proteins (e.g., fibrin or collagen). The culture chamber can be flanked by another chamber that may contain a chemoattractant. While flow through the chamber continuously supplies media and cytokines, gradients form between the flanking chambers in the direction perpendicular to the flow (Figures 6F,G). Finite element analysis and numerical solutions to the convection-diffusion equation have been used to predict solute distributions. Computational predictions have been validated *in vitro* with fluorescent molecules that have diffusive properties similar to VEGF such as conjugated dextran (Barkefors et al., 2008; Shamloo et al., 2008; Vickerman et al., 2008; Chung et al., 2009; Huang et al., 2015; Shirure et al., 2017) and AlexaFluor 488 (Zengel et al., 2011). A major advantage of these microfluidic devices is that they facilitate time-series imaging.

Barkefors et al. designed a single channel microfluidic device to study the rate of chemotactic growth along a hill-shaped diffusive gradient (Barkefors et al., 2008). HUVECs or human umbilical artery ECs (HUAECs) were cultured with two VEGF splice variants VEGF_165_, VEGF_121_, or FGF-2 supplemented ubiquitously or as a gradient and imaged every 5 min for 200 min. Chemotaxis and chemokinesis effects were delineated by measuring the net distance of migration toward the growth factor (chemotaxis) and the total cell migration distance (chemokinesis). Both VEGF splice variant gradients induced chemotaxis, but not chemokinesis. HUVECs were slightly influenced by FGF-2 chemotaxis, but HUAECs were not affected, suggesting that ECs from different origins may exhibit different behavior. They also noted that cells near the peak concentration migrated negligibly compared to regions of sharper gradients, indicating that cells near growth factor sources become non-migratory. A follow-up study investigated the role of FGD5, a guanine nucleotide exchange factor found in endosomes containing VEGF receptor 2 (Heldin et al., 2017). HMVECs were analyzed in the same microfluidic device used by Barkefors et al. or were transfected with siRNA to knockdown FGD5. HMVECs lacking FGD5 were found to have reduced chemotactic response to VEGF gradients and accelerated VEGFR2 degradation and signaling.

Fibrous matrices have been included in microfluidic assays to better represent soft tissue mechanics and facilitate sprouting chemotactic studies. Shamloo et al. adhered ECs to microbeads embedded in a collagen-fibronectin mix in the cell culture channel of their microfluidic device (Shamloo et al., 2012). A VEGF gradient was established across the culture chamber and growth was imaged daily. Time-lapse images showed initial EC organization into sprouts, which later grew from the bead surface into the collagen-fibronectin matrix and finally up the VEGF gradient. By adjusting the collagen concentration and magnitude of the VEGF gradient, they observed that denser matrices (1.9 mg/ml) impaired alignment of sprouting cells along the VEGF gradient. However, alignment of sprouts was observed in lower ECM density (1.2 mg/ml). Notwithstanding, alignment could be achieved at lower densities without changing VEGF gradient, but increasing minimum VEGF concentration. This suggests that both cytokine gradient and concentration influence microvessel migration. Furthermore, sprout trajectories were tracked over time to determine how quickly cells aligned along the VEGF gradient. Sprouts in denser matrices took more time to align than sprouts cultured in less dense matrices. The time for an initial sprout to form and grow from the microbead also increased with increasing matrix density. In a follow-up study, daily images informed metrics such as the number of cells per sprout, sprout length, thickness, and elongation rate over time (Shamloo et al., 2016). This information was used to create a computational model of sprouting angiogenesis that was validated with time-lapse data.

In addition to gradients formed from cytokine supplemented media, gradients can be established *via* coculture. Vickerman et al. stimulated cellular migration first along synthesized recombinant cytokine gradients then along coculture-derived cytokine gradients in the same device (Vickerman et al., 2008; Takuwa et al., 2010). For the first study they established a gradient of sphingosine-1-phosphate (S1P) – a lipid mediator associated with EC proliferation, migration, lumen formation, and endothelial barrier function (Takuwa et al., 2010). Time-lapse imaging of migrating HMVECs demonstrated filopodial extension and retraction, chemotaxis along S1P gradients, and lumen formation (Vickerman et al., 2008). For the second study, the source channel was seeded with MTLn3 cancer cells, U87MG cancer cells, or smooth muscle cells to serve as a source of cell-secreted growth factors. MTLn3 cells induced chemotaxis at a rate lower than VEGF alone while U887MG failed to induce chemotaxis. By contrast, SMC coculture reduced migration and had a slight negative chemotactic effect on HMVECs in which cells migrated away from the source of cytokines. They also investigated the timing of VEGF administration on chemotaxis. Migration was strongest when VEGF gradients were established 1 day after seeding HMVECs and was reduced when the VEGF gradient was introduced after 3 days (Chung et al., 2009).

In addition to custom microfluidic devices, a few commercial options have emerged such as the μSlide chemotaxis assay (Zantl and Horn, 2011). In one coculture time-lapse study using this assay, HUVECs were cultured with VEGF producing carcinomic FaDu cells. Surprisingly, adding supernatant from FaDu cells to the chemoattractant source reservoir did not induce chemotaxis, but culturing FaDu cells in the reservoir elicited chemotaxis (Zengel et al., 2011). In another time-lapse study using the μSlide assay, HUVECs migrated toward a Wnt3a gradient while knockdown of Kif26b, a kinesin associated with microtubules, lead to longer and more scattered trajectories. These results provided evidence that Kif26b interacts with disheveled 3 (Dvl3) Daam1 complexes that could regulate microtubule stability during the formation of polarized cell edges during angiogenesis (Guillabert-Gourgues et al., 2016).

Microfluidic-based approaches can be optimized to reduce confounding variables. Further, precise gradient control improves reproducibility during time-series evaluation of chemotaxis. Custom and commercial microfluidic devices have enabled simple studies that accommodate diverse *in vitro* culture models and improve on the Boyden bridge chamber methods by easing gradient quantification and providing long-term gradient stability.

### Assessment of Chemotaxis by Matrix-Bound Cytokines

Cytokine splice variants have different binding affinities for matrix proteins, which can result in unique spatial distributions of each splice variant (Vempati et al., 2011). For example, the longer isoforms of VEGF, VEGF_165_, and VEGF_189_ can be sequestered by heparan sulfate proteoglycans (HSPGs), while VEGF_121_ lacks the binding domain contained by the longer isoforms. Investigators have exploited this effect to generate gradients in cell culture. For instance, Liu et al. electrochemically immobilized gradients of fibronectin, VEGF_165_, or both onto slides (Liu et al., 2007). The gradient profiles ranged between the hill and step profiles. All gradients triggered an increase in EC migration, with VEGF gradients causing more migration than fibronectin. Combining gradients resulted in a larger increase in migration, indicating that both growth factors and the ECM affect cellular migration, and their integration can be additive.

Approaching matrix-bound chemotaxis from a different angle, Li et al. investigated the role of cell surface receptors (Li et al., 2016). Syndecan-4 (Synd4) is a heparan sulfate proteoglycan (HSPG) cell surface receptor that has previously been implicated in haptotaxis during angiogenesis (Bass et al., 2007) as well as chemotaxis due to its ability to act as a co-receptor and independent receptor for FGFs. HUVECs were transfected with null and knockout genes for Synd4 then seeded in μSlide chemotaxis assays. A locally linear bFGF gradient was introduced and imaged every 30 min for 18 h. There was a linear relationship between the initial position of a cell along the gradient and the accumulated distance the cell moved, allowing prediction of the distance a cell would travel based on its initial position. Synd4 knockdown was associated with reduced accumulated distance and Euclidean distance, although there was no difference in proliferation (Elfenbein and Simons, 2013).

## Stromal Cell Interactions

**Figure 7:**
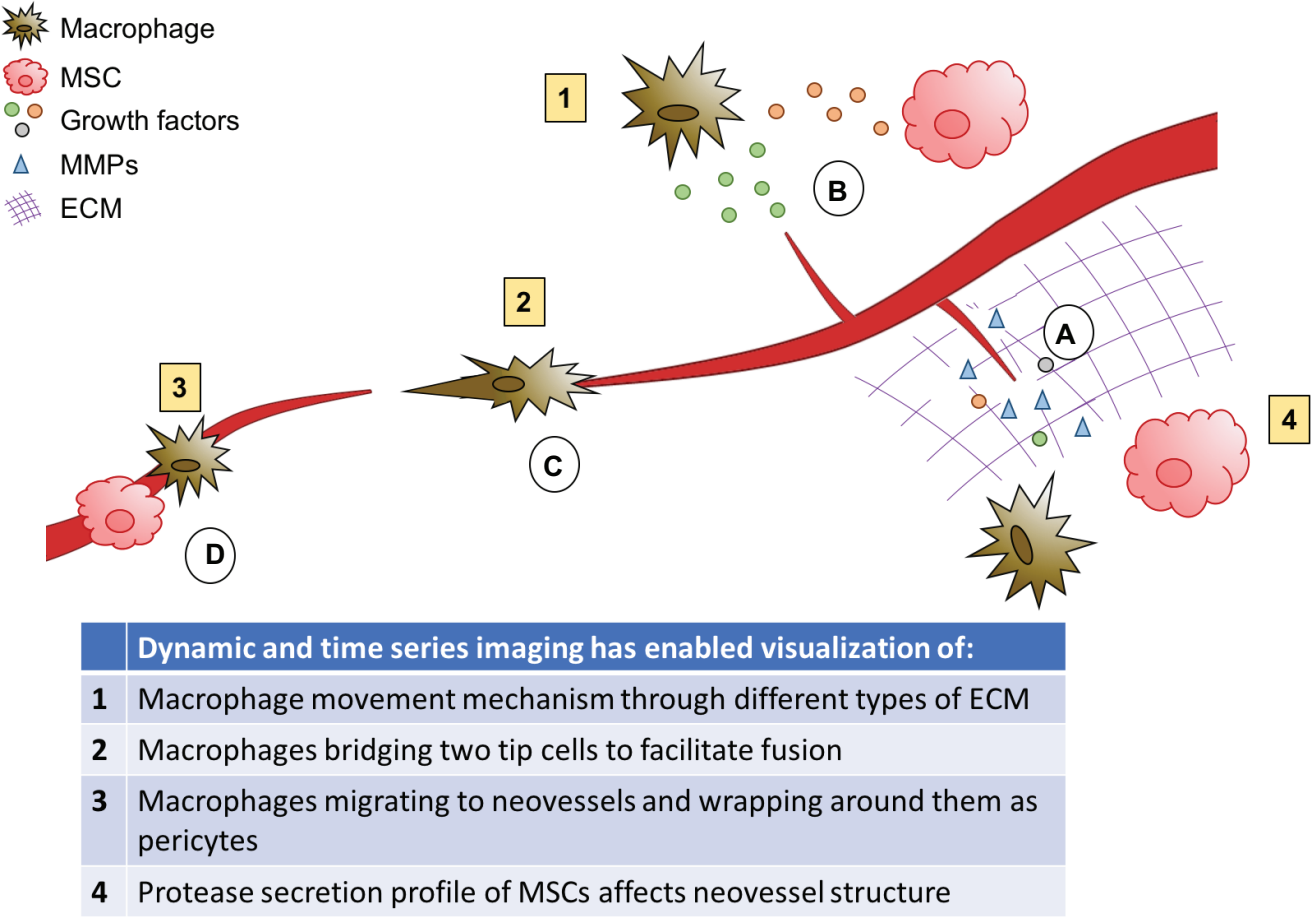
Role of stromal cells in angiogenesis. Macrophages and MSCs secrete MMPs, which degrade the matrix to allow sprouts to form and grow, and releases matrix bound growth factors. **(A)** Macrophages and MSCs secrete growth factors to stimulate sprouting and guide growing neovessels, and MSCs secrete factors to attract macrophages and modulate their phenotype. **(B)** Macrophages can facilitate neovessel fusion by bridging two tip cells. **(C)** Macrophages and MSCs can also act as pericytes to stabilize newly developed blood vessels. **(D)** MSCs also aid in lumen formation.

Stromal cells are a broad classification of cells located in the ECM surrounding the microvasculature, including fibroblasts, pericytes, macrophages, MSCs, and others. Macrophages and MSCs play critical roles throughout angiogenesis, interacting directly with both microvessels and their surrounding matrix to facilitate sprouting, growth, vessel fusion, and tissue remodeling. The role of stromal cells in angiogenesis is shown graphically in Figure 7. Here, we will discuss the dynamic interactions of these cells with microvessels and matrix through all phases of angiogenesis, and how different imaging techniques have increased our understanding of the temporal behavior of these cells.

### Macrophages

While the spectrum of reported macrophage phenotypes has broadened considerably in recent years, macrophages are often discussed in terms of two, general phenotypes related to inflammation and tissue repair: the “classically activated” M1 and “alternatively activated” M2 phenotypes. M1 macrophages are strongly present in regions of inflammation and have historically been considered anti-angiogenic (Kodelja et al., 1997; Jetten et al., 2014). However, recent reports suggest they may directly influence early stages of angiogenesis (Spiller et al., 2014; Gurevich et al., 2018). The M2 phenotype is more typically associated with healing and angiogenesis (Kodelja et al., 1997; Jetten et al., 2014). These two general phenotypes can be broken down into a spectrum of sub-phenotypes with distinct chemokine profiles (reviewed in Mantovani et al., 2004). Assessing the spatial and temporal role of different macrophage phenotypes and sub-phenotypes is critical for understanding and controlling angiogenesis.

Spiller et al. compared the effects of M0 (unactivated), M1, M2a, and M2c macrophages over time during angiogenesis, using traditional *in vitro* sprouting assays and *in vivo* scaffold implantation (Spiller et al., 2014). They found that M1 macrophages may play a critical role in early stages of angiogenesis by secreting VEGF, FGF, and other growth factors to stimulate neovessel sprouting, and M2c macrophages secreted high levels of MMP-9. MMPs locally degrade the basement membrane of the parent vessel, enabling sprout formation. M2a macrophages then secrete PDGF to recruit pericytes and MSCs to stabilize vessels, and TIMP3 to halt the effects of M1 macrophages (Spiller et al., 2014). When scaffolds were implanted in mice, they found that incorporating both M1 and M2 phenotypes were necessary for vascularization, although the balance of each phenotype could not be quantified during early and late stages of neovessel growth (Spiller et al., 2014). Still, this study demonstrates the critical role of macrophage phenotypic shifts throughout angiogenesis.

While this information is a critical step toward understanding the spatiotemporal role of macrophage phenotype, an inability to perform time-series analyses in these mouse studies limited potential insight. Because animals must be sacrificed at each time point, the same sample cannot be evaluated continuously over time. Thus, it is difficult to determine if and when macrophage phenotypic changes are occurring in the tissues. Techniques that allow for dynamic analysis of samples over time may be key to unlocking the mechanisms driving macrophage behavior and phenotypic transition over time during angiogenesis.

Zebrafish eggs have become a popular tool for studying angiogenesis with dynamic imaging. Because zebrafish eggs are clear, live imaging techniques can be used to examine how microvessels and macrophages, or other stromal cells, behave over time during development. Gurevich et al. utilized zebrafish eggs from a transgenic fish line with fluorescent macrophages and blood vessels to study macrophage behavior following injury (Gurevich et al., 2018). They observed that M1 macrophages were most predominant at the sprouting stage of angiogenesis, where they localized at neovessel tips. Non-M1 macrophages had a higher presence in mid-late stage angiogenesis, and associated more randomly with vessels, sometimes wrapping around the vessel similarly to pericytes. At the remodeling stage, macrophages appeared to reduce the number of neovessels by inducing endothelial apoptosis, which is necessary after the damaged tissue has healed and metabolic demands are decreased. A decrease in M1 macrophages is critical for this remodeling (Gurevich et al., 2018). Overall, M2 macrophages appeared to have less of an effect on neovascular density than M1 macrophages (Gurevich et al., 2018), which is contrary to earlier studies (Kodelja et al., 1997; Jetten et al., 2014). It is possible that these discrepancies over the effects of M1 macrophages may be due to further sub-phenotype differences within both M1 and M2 macrophages that may have varied between studies. In this case, live imaging was able to confirm the results of previous studies, and establish a more precise timeline and deeper understanding of macrophage movement and behavior.

Live imaging has also enabled researchers to gain an understanding of how macrophages are moving through the extracellular matrix. Van Goethem et al. observed macrophage migration in both fibrillar and non-fibrillar collagen matrices using a 3D *in vitro* migration assay (Van Goethem et al., 2010). They observed that macrophages perform amoeboid movement through fibrillar collagen, which is when the cell extends protrusions forward and “crawls” through the matrix using pseudopodia. When cultured in Matrigel or non-fibrillar gelled collagen, the macrophages adapted their movement to mesenchymal, meaning that the cell extends a larger protrusion forward, secretes proteases to degrade the extracellular matrix, and creates a path through which it can travel. This was an important observation, as it indicates that macrophages can adapt their migration mechanism to move through different tissue types throughout the body (Van Goethem et al., 2010).

Live imaging has also been used to study macrophage movement *in vivo* in Takeda fish. Grabher et al. observed that macrophages become more motile as they mature, switching from a rounded shape typical of amoeboid migration to a flattened phenotype with long protrusions as their motility increased (Grabher et al., 2007). When the PI3K signaling pathway was inhibited, the authors observed reduced macrophage migration, suggesting it plays a critical role in macrophage movement (Grabher et al., 2007).

Macrophages are critical for the process of forming vessel intersections. Fantin et al. observed the developing mouse brain and found that macrophages appear to bridge two tip cells growing toward each other just prior to fusion (Fantin et al., 2010). The authors then used live imaging of zebrafish to confirm the role of macrophages in this process. They were able to observe in real-time that macrophages migrate to areas of vessel fusion and spread between two vessels, interacting with both tip cells until they fused (Fantin et al., 2010). However, the exact mechanisms for vessel fusion, and the role of macrophages, remain to be determined. A similar effect was observed in a zebrafish model of brain vascular ruptures, where live imaging enabled the visualization of macrophages extending their filopodia to form physical contacts between two disconnected endothelial cells and pulling them together (Liu et al., 2016). It is possible that a similar mechanism is causing two tip cells to come together during the fusion of two growing neovessels.

Macrophages play distinct roles in angiogenesis by secreting cytokines and degrading the ECM to enable sprouting; they guide neovessel growth, recruit stabilizing cells, facilitate vessel fusion, and trigger apoptosis to remodel tissues. Macrophage phenotypic transition may play a critical role in moving from one stage of vessel growth to the next, as each phenotype and sub-phenotype is activated by and produces a different combination of signaling factors. It has also been suggested that matrix degradation products can trigger a phenotypic switch from M1 to M2 phenotype (Sicari et al., 2014). However, further understanding is still needed of the environmental factors that trigger macrophage phenotypic transition and the role of each sub-phenotype on angiogenesis. Future advancements in live imaging to track macrophage phenotypic changes may help answer some of these key questions.

### Mesenchymal Stem Cells

MSCs are a type of stromal cell found in several different tissues. They can differentiate into a variety of cell types, including smooth muscle cells and pericytes. MSCs are known to have a broad range of tissue healing functions, including a critical role in angiogenesis. They secrete growth factors, such as VEGF, PDGF, and Ang-1, to stimulate angiogenesis (Chen et al., 2008). MSCs also recruit and activate macrophages and modulate macrophage phenotype (Chen et al., 2008; Nakajima et al., 2012; Cho et al., 2014; Wise et al., 2014). Like macrophages, they secrete MMPs, which are critical for MSC-mediated vessel sprouting, neovessel assembly, and tissue remodeling (Collen et al., 2003; Chun et al., 2004; Ghajar et al., 2006, 2010; Kachgal and Putnam, 2011; Wise et al., 2014).

Kachgal-Putnam et al. showed in an *in vitro* model that MSCs from different tissue types, specifically bone marrow and adipose tissue, secrete different profiles of proteases to modulate angiogenesis (Kachgal and Putnam, 2011). Bone marrow-derived MSCs primarily secrete MMPs, while adipose-derived MSCs secrete more plasmin. This suggests that the protease secretion profile of MSCs may be specific to the ECM compositions of different tissues, to best support neovessel growth through the matrix. They also found that selectively inhibiting different proteases affects neovessel phenotype. Inhibiting MMPs resulted in distended vessels, although it did not affect vessel length. This suggests that MSC protease secretion profile may play a role in determining vessel phenotype and structure in different tissues (Kachgal and Putnam, 2011). Others have shown that MMPs play a larger role in angiogenesis, halting EC tube formation altogether if inhibited (Collen et al., 2003; Ghajar et al., 2010). These discrepancies suggest that other environmental factors may be playing a role in MSC-mediated angiogenesis that are not yet understood.

Further studies are needed to characterize the complex interactions between the cell types in the stroma and the microvascular network. *In vitro* studies do not sufficiently replicate the dynamic multi-cellular *in vivo* environment. However, studying angiogenesis in animals can be challenging, due to the need to sacrifice animals at each time point, the reproducibility, and the decreased access to imaging. The ability to track the same sample over time may allow for a more accurate understanding of the progression of angiogenesis. Above, we discussed transparent zebrafish as a tool for acquiring live videos of angiogenesis. Bioluminescence imaging is another alternative that allows for *in vivo* imaging of an anesthetized animal. Implanted cells can be engineered to contain luciferase, a protein that will emit light when activated. Luciferase can be activated at multiple time points by injection of luciferin, enabling time-series imaging of a single sample following implantation.

**Figure 8:**
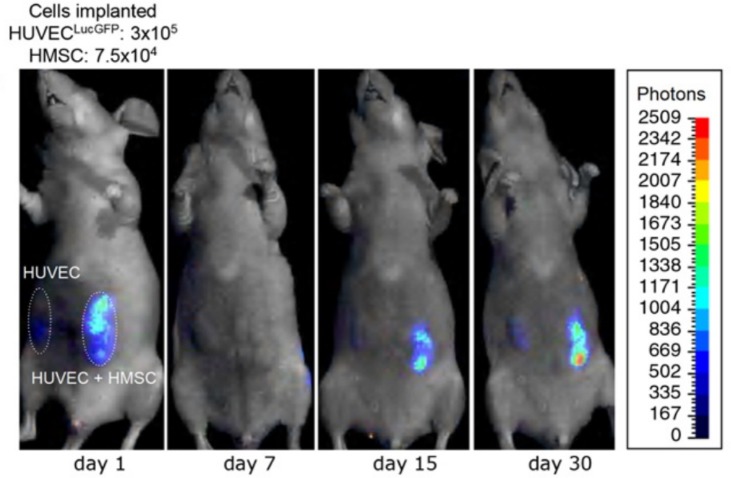
Time-lapse bioluminescence imaging of rats with implanted HUVECs or HUVECs + HMSCs. After an initial drop, the number of luciferin-activated cells increases over time to a greater extent in the HUVEC + HMSC group compared to the HUVEC only group. This suggests increased vascular network formation in the implant with HMSCs, compared to HUVECs alone. This figure was reproduced with permission from Elsevier (Sanz et al., 2008).

Sanz et al. used bioluminescent imaging to monitor the formation of vascular networks in mice (Figure 8; Sanz et al., 2008). They implanted scaffolds with HUVECs (expressing luciferase) alone or HUVECs and MSCs together and imaged the constructs periodically for 120 days. Constructs with MSCs, most notably at later time points, measured a higher number of photons per second, indicating that a higher number of HUVECs were exposed to the luciferin in those samples. It is difficult to determine if this was due to the presence of a more mature vascular network, exposing more of the construct to the luciferin, or if there were simply changes in the number of HUVECs. The authors used other methods, including histological and immunohistochemical analysis, to verify that more mature vascular networks (vessels containing erythrocytes) were the cause of the increased bioluminescent signal in scaffolds containing MSCs (Sanz et al., 2008). Using bioluminescent imaging in live mice, others have demonstrated that MSCs enhance vascular network formation (Roura et al., 2012; Todeschi et al., 2015). While bioluminescent imaging is a useful tool for establishing time courses of increased vascularization, it is limited in its ability to evaluate vessel structure and function.

MSCs and macrophages are key examples of stromal cells that are involved in multiple stages of angiogenesis. Due to their spatiotemporal roles throughout angiogenesis, they are ideal targets for dynamic imaging. However, other cell types may also benefit from this approach. Pericytes are recruited during angiogenesis to wrap around the endothelium and stabilize the neovessel, and have also been shown to enhance microvessel elongation and directed migration using time-lapse imaging (Arima et al., 2011). In tumors, cancer-associated fibroblasts also play a role in stimulating angiogenesis by secreting angiogenic cytokines. Dynamic imaging methods may provide insights into the behavior of these other cell types as well.

## Discussion

This review highlights how time-lapse imaging techniques have advanced our understanding of the interaction of blood vessels with their environment during sprouting angiogenesis. Angiogenesis is dynamically influenced by a variety of environmental factors of the surrounding tissue matrix, as vessels interact with the ECM. Hence, the role of the ECM is vast and includes biophysical, mechanical factors, and chemotactic factors. The imaging techniques have provided the means to observe, characterize, and quantify the evolution of microvessel behavior, providing new insights on the process of angiogenesis and the interaction of microvessels with the ECM. Thus, data derived from time-lapse imaging experiments holds great promise to further elucidate angiogenic responses and advance the field. For example, the information could be observation and quantification of multiple signals experienced by microvessels. This will help determine how signals are integrated to promote a functional microvascular network, a key problem of angiogenesis (Ingber et al., 1995). It could also help bridge our understanding between extracellular signals and intracellular events such as EC metabolic pathways (Eelen et al., 2018), and EC phenotypic behaviors such as tip cell overtaking (Jakobsson et al., 2010; Arima et al., 2011). Lastly, as time-series imaging techniques improve to *in vivo* in mammals, we could gain better insight into angiogenic invasion in tumors, treatment efficacy, and graft acceptance.

**Figure 9:**
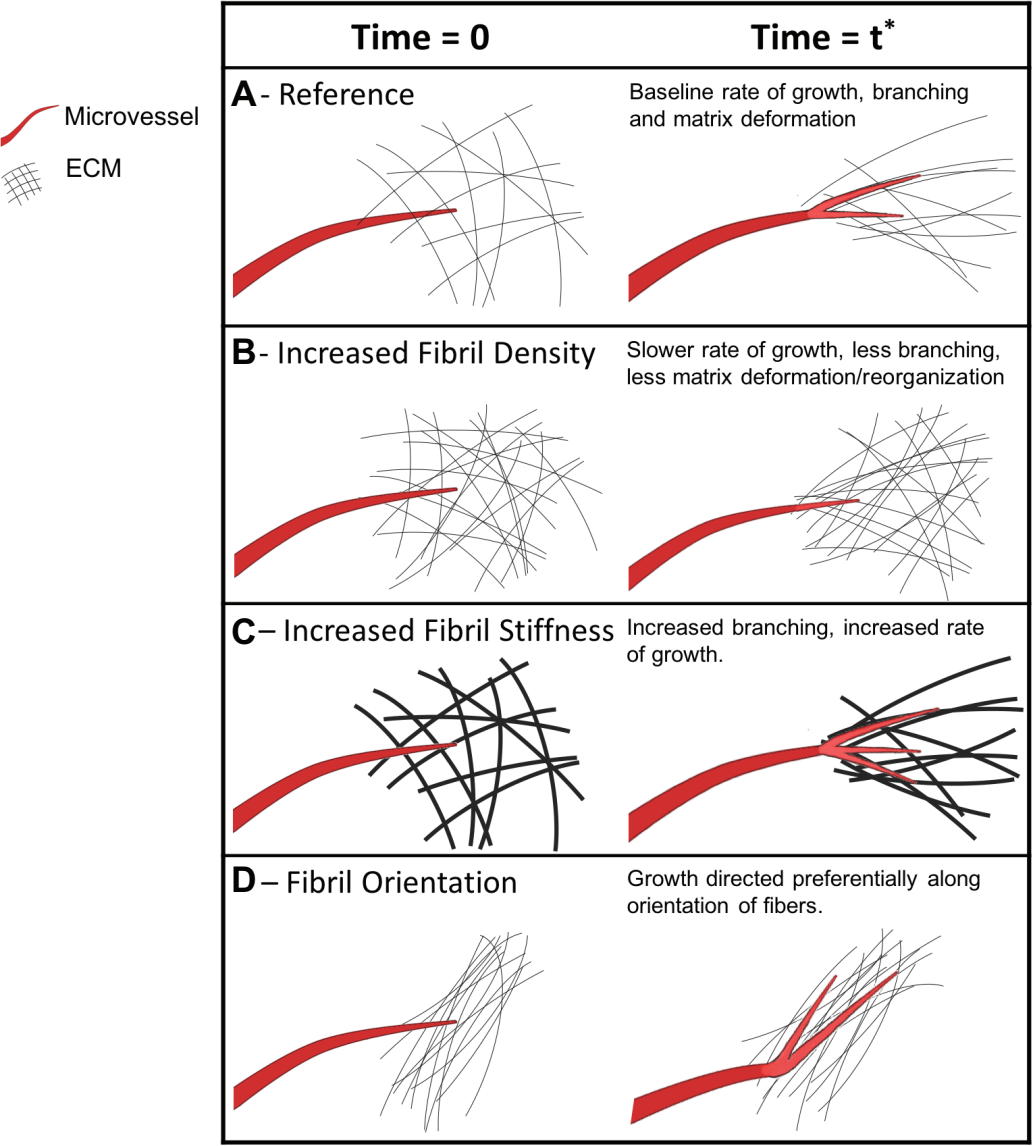
Mechanical and biophysical signals of a microvessel as it interacts with its microenvironment during growth. Microvessels are shown in red, new microvessel growth is shown in brighter red on the right column, and extracellular matrix fibers are shown in black. The first column of each section shows an arbitrary initial point in time when a vessel is first observed. The vessel remains the same across the rows but ECM conditions are altered. The second column represents a later point in time of the same vessel and its ECM microenvironment. **(A)** A microvessel forms cell-ECM adhesions, exerts pulling forces, and deforms the ECM during growth. **(B)** Higher ECM density, along with increased stiffness, causes microvessels to induce decreased deformation and decreased growth. **(C)** Stiffer ECM independent of fibril density leads to increased branching. **(D)** Pre-existing ECM fiber alignment induces directed neovessel growth.

Despite advances in our understanding of the biophysical and mechanical interaction of angiogenic microvessels with the ECM (Figure 9), there are a number of areas that warrant further investigation. The stiffness, density, and microstructure of the ECM, and forces exerted by microvessels alter angiogenic behavior (Krishnan et al., 2007; Han et al., 2018). However, most of the available experimental data focuses on global measures of vascular network morphometry. Little is known about the effects of local perturbations in biophysical properties on microvessel behavior. For instance, the *in vivo* mechanotransductive signaling cascades that lead to deterministic vessel behavior by nonlinear integration of biophysical signals remain unknown. However, other studies that did not investigate angiogenesis have introduced techniques to measure local ECM stiffness and focal adhesion complexes using time-lapse imaging (Han et al., 2018; Kang et al., 2018). The gap in our knowledge about microvessel interaction with its environment stems from lack of experimental data on the local interaction of microvessels with their stromal environment and capturing the dynamic interplay over time. This information could be provided by experiments utilizing time-series imaging, and these data are necessary to advance our understanding of angiogenesis.

The role of chemotaxis in angiogenesis has been only partly understood, as traditional chemotaxis assays were unable to describe cellular migration, vessel elongation, or the development of the microvascular network. Time-series imaging techniques helped distinguish tip and sprout behavior in the absence of autocrine gradients (Gerhardt et al., 2003), and established on what day of culturing VEGF gradients were most influential on cell (Chung et al., 2009). Microfluidic devices and computational methods have enabled the precise control and quantification of soluble and matrix-bound cytokine gradients. The methods could potentially be modified to incorporate multicellular sprouting assays that recapitulate angiogenesis more closely. This would enable a more holistic study of angiogenesis by accounting for intercellular interactions and tip-stalk cell dynamics. In conjunction with time-lapse imaging, these approaches could advance understanding of the effects of spatiotemporal cytokine gradients on sprouting microvascular networks.

The effects and role of protease activity during angiogenesis have been largely discovered, yet the exact role of proteases over time and space is not well understood. The available methods based on fluorogenic substrates show great promise, but they have seen limited use in the study of angiogenesis to date. There are some notable limitations of these techniques, however. The two common approaches to their application have been to include a fluorogenic enzyme substrate either during polymerization of a surrogate ECM such as collagen and Matrigel or as an additive to a culture containing proliferating ECs or angiogenic microvessels. In the former approach, fluorescence from the substrate continues to increase over time in culture and at some point, and becomes the limiting factor for performing time series imaging over longer periods. In the case of spiking the culture, the approach relies on diffusive transport of the fluorogenic substrate throughout the culture. There is a time constant associated with this diffusive process, which can limit temporal resolution and, thus, utility of the approach. In both cases, fluorescence produced by protease activity on a fluorogenic substrate represents a “high water mark” of protease activity, as the fluorescent signal continues to accumulate over time. Thus, there is certainly room for improved techniques for times-series imaging of protease activity.

Another key problem of angiogenesis is determining the role of stromal cells to facilitate sprouting, growth, vessel fusion, and tissue remodeling. Stromal cells are essential for many angiogenic processes, and dynamic imaging has helped to uncover the interaction of stromal cells with both microvessels and their surrounding matrix. Dynamic imaging has provided knowledge about more precise macrophage phenotype activity throughout the timeline of angiogenesis (Gurevich et al., 2018), macrophages migration mechanisms by tissue type (Grabher et al., 2007, p. #81; Grabher et al., 2007; Van Goethem et al., 2010), the role of macrophages in vessel fusion (Fantin et al., 2010; Liu et al., 2016), enhanced vascular network formation and maturation in the presence of MSCs (Sanz et al., 2008). However, improved technology is needed to visualize the real-time behavior of MSCs throughout angiogenesis *in vivo*. Imaging *in vivo* is particularly important, as *in vitro* experiments as of yet do not replicate the complexity of the *in vivo* environment. Angiogenesis is a complex process that relies on communication between multiple cell types and vessels, secretion of precise profiles of chemokines and proteases, and the controlled degradation of the extracellular matrix. Advancements in real-time imaging may help understand the complex interplay between these components during different stages of angiogenesis. Bioluminescence imaging has enabled time-series imaging of mammals *in vivo* without the need for sacrifice. However, it has a limited scope and can only be used for indirect quantification of vascular network development. While this technology is a major advancement, we are still unable to visualize specific cellular behaviors over time in mammals. Technologies addressing this limitation have enormous potential to advance our understanding of cellular behavior throughout angiogenesis. Moving forward, more work is needed toward understanding the complex relationship between stromal cells and other factors that support angiogenesis, such as mechanical, biophysical, and biochemical stimuli. Imaging methodologies that allow us to monitor stromal cell behavior along with ECM biophysical, mechanical, and chemotactic factors would provide a more comprehensive understanding of angiogenesis.

## Author Contributions

All authors contributed to the conception and writing of this review article.

### Conflict of Interest Statement

The authors declare that the research was conducted in the absence of any commercial or financial relationships that could be construed as a potential conflict of interest.
